# The Sound of Fear: How Horror Soundtracks Shape Threat Perception of Emotional Faces

**DOI:** 10.1177/17470218261447279

**Published:** 2026-04-25

**Authors:** Li Fun Lua, Xuan Nee Kong, Jasmine K. W. Lee, Shumetha Kaur Sidhu

**Affiliations:** 1 School of Psychology and Clinical Language Sciences, University of Reading Malaysia, Johor, Malaysia; 2 School of Psychology and Clinical Language Sciences, University of Reading, Berkshire, UK

**Keywords:** scary music, threat perception, emotion, affective priming

## Abstract

Music strongly shapes emotional perception, yet its influence on social threat evaluation remains underexplored. This study examined whether scary music biases threat perception of emotional faces using subjective ratings and pupillometry. In a within-subjects design, a predominantly female sample of participants viewed angry, neutral, and happy faces following either scary music or white noise. Behavioural ratings showed that faces were perceived as more threatening after scary music relative to white noise, consistent with affective priming accounts. Notably, the effect extended beyond angry and neutral faces to happy faces, suggesting that the pairing with fear-inducing music distorted their positive meaning, perhaps conveying ill intent instead. Pupillometry results revealed no overall effect of scary music on pupil size; however, an interaction showed increased pupil dilation to neutral faces under scary music, consistent with the heightened susceptibility of ambiguous stimuli to priming. Angry and happy faces may have elicited sufficient arousal on their own, limiting additional music-related effects. These findings demonstrate that scary music biases threat perception both behaviourally and physiologically, though not uniformly across emotions. By showing that music can alter the evaluation of socially meaningful stimuli (i.e., facial expressions), this study advances understanding of cross-modal affective priming and has implications for contexts where music may unconsciously shape social judgements.

## Introduction

Music plays a significant role in many people’s lives, often accompanying a variety of activities in different settings. With the advancement of portable music technology such as smartphones, music has become more accessible than ever before, making it a more constant presence in everyday life. Given the prevalent presence of music in daily life, it is important to explore how listening to music can affect one’s emotional state and perception of the world.

Often referred to as “the language of emotion,” music has the powerful ability to express and evoke feelings, both for the musician and the listener (Corrigall & Schellenberg, 2013, p. 299). It acts as a universal medium through which one’s feelings can be communicated and perceived, with research showing that basic emotions in music can be recognised cross-culturally (Fritz et al., 2009). These emotions arise from various mechanisms such as associations with real-life events and experiences (Juslin & Laukka, 2004) or the combination of acoustic features such as tempo, pitch, and dissonance (Kallinen & Ravaja, 2006). For example, fast tempos and major keys are commonly associated with happiness and excitement, whereas slow tempos and minor keys tend to evoke sadness (Hunter et al., 2010).

This use of emotional cues and acoustic features is especially evident in horror films, where soundtracks frequently utilise unstable pitches, trembling notes, and rapid key changes to instil fear and build suspense (Blumstein et al., 2010; Brownrigg, 2003). Such music aims to manipulate the audience’s emotional state, potentially making certain movie scenes appear scarier. Researchers also report that horror movie soundtracks frequently utilise noises resembling human screams (Trevor et al., 2020), further contributing to their effectiveness at eliciting fear and anxiety. These scream-like sounds are emotionally salient and engage subcortical pathways – including the amygdala – that monitor the environment for significant cues (Arnal et al., 2015; Aubé et al., 2015; Kong & Zweifel, 2021). By triggering amygdala activity, scream-like sounds in horror soundtracks may function as highly salient stimuli, biasing attention towards threat-relevant information and preparing the body for adaptive responding.

This engagement of the autonomic nervous system manifests in measurable physiological changes associated with arousal and heightened attention (Koelsch et al., 2013; Pannese et al., 2016; Rodrigues et al., 2023), such as pupil dilation (pupillometry; Einhäuser, 2017; Grujic et al., 2024). When individuals engage with emotionally arousing stimuli, the sympathetic branch of the autonomic nervous system is activated, leading to an increased activity of the dilator muscles responsible for pupil dilation (Steinhauer et al., 2004). Although amygdala activity is not measured in the present study, prior work on salience processing provides a useful framework for understanding how auditory cues may influence autonomic arousal. For example, in a study by Ansani et al. (2020), participants’ eyes were tracked as they viewed a movie scene paired with either sad or anxiety-inducing music. Those who listened to anxiety-inducing music showed greater pupil dilation and vigilance, suggesting heightened arousal and sensitivity to salient cues. However, pupil size can also be influenced by low-level visual factors such as luminance, contrast, or spatial frequency (Kim et al., 2023; Oster et al., 2022). Therefore, it is crucial to acknowledge and control for these factors to ensure that the observed effects reflect cognitive efforts and processes, rather than visual confounds.

The effect of music on perception can be further explained by affective priming, where the emotional content of a stimulus affects the perception and interpretation of a subsequent stimulus (Klauer & Musch, 2003). In this context, emotion-inducing music may act as an affective prime, establishing an emotional context that shapes how subsequent stimuli are perceived. Supporting this, several studies have explored the influence of emotion-inducing music on the emotional perception of visual stimuli. For instance, Logeswaran and Bhattacharya (2009) observed that music influenced the perceived emotional intensity of facial expressions: happy faces were rated as happier, and sad faces as sadder, when the faces were paired with the emotionally congruent music (happy or sad). Neutral faces were also perceived as happier with happy music and sadder with sad music, suggesting that participants may use the emotional context from the music to interpret emotionally neutral expressions

Similarly, Prinz and Seidel (2012) found that exposure to fear-inducing music biased participants’ perception of ambiguous images. Participants who listened to scary music were more likely to interpret a visually ambiguous image as threatening, whereas participants exposed to no music or happy music primarily reported harmless interpretations. Together, these studies suggest that emotional music can bias the interpretation of visual stimuli, especially for ambiguous stimuli (Logeswaran & Bhattacharya, 2009; Prinz & Seidel, 2012).

Notably, there appears to be a lack of studies investigating how scary music affects the perception of threat in faces. Existing work has largely focused on music and emotional perception (Logeswaran & Bhattacharya, 2009) or on threat perception of visually ambiguous objects (Prinz & Seidel, 2012), but little is known about the interaction between music and threat perception of social stimuli. Addressing this gap is important to gain a better understanding of how scary music can bias the threat perception of faces, and whether its effects vary depending on the displayed emotion. Since facial expressions play a big role in facilitating social communication (Jack & Schyns, 2015), misinterpreting someone’s emotional expressions (e.g., perceiving a face as more threatening than it actually is) could have implications for various real-world contexts, such as clinical and legal settings, or other environments where emotion perception influences decision-making.

Thus, this study investigates how scary music influences the perception of threat in emotional faces by examining subjective behavioural ratings and pupil dilation. Pupillometry provides a noninvasive measure of pupil-linked arousal that reflects activity of the locus coeruleus–noradrenergic system and is sensitive to changes in autonomic arousal, cognitive effort, and salience (Viglione et al., 2023). Although pupil dilation is not specific to threat processing per se, it has been shown to reliably increase in response to emotionally and motivationally salient stimuli, including threat-related cues (Ansani et al., 2020; Finke et al., 2021; Snowden et al., 2016). By presenting angry, happy, and neutral faces after different auditory conditions, we aim to explore whether exposure to scary music influences perceived threat in various facial expressions. In this study, the term “scary music” refers to music designed to evoke feelings of fear and threat by deliberately utilising specific musical features such as unstable pitch, unpredictable volume changes, and harsh, scream-like sounds (Blumstein et al., 2010; Trevor et al., 2020).

Considering previous research, our hypotheses were as follows: When comparing the scary music condition to the white noise condition, we expected to see (a) higher threat ratings specifically for angry and neutral faces, with little or no change for happy faces, and (b) larger pupil sizes in response to faces of all emotions.

## Methods

### Participants

Forty-five participants were recruited for the study (a predominantly female sample, with only three males), aged between 18 and 25 years old (*M* = 20.91, *SD* = 1.56). The sample included 20 Chinese, 13 Malay, 6 Indian, and 6 participants of other ethnicities. Sample size was determined a priori with G*Power 3.1.9.7 (Faul et al., 2007), using an effect size of η_*p*_^2^ = .16 obtained from a previous study by Prinz and Seidel (2012), which suggested a minimum sample size of 43 participants.

This study was approved by the Research Ethics Committee of the authors’ institution. Participants were eligible for the study if they were university students aged 18 years and above (as of their last birthday) with sufficient English comprehension and self-reported normal or corrected-to-normal vision and hearing.

### Apparatus and Materials

Stimulus presentation and data collection were programmed in Tobii Studio 3.4.5. Pupil sizes (a physiological index of autonomic arousal and/or salience) were recorded binocularly with the Tobii X2-60 Eye Tracker at a sampling rate of 60 Hz. Participants sat 60 cm away from the monitor, which produced a resolution of 1.0 arcminute per pixel. All participants performed a 9-point calibration before the experimental tasks. To control for visual confounds, a standard monitor setup with fixed overhead lighting was used. Over-ear headphones (volume fixed at 24%) were used to control for volume and external noise during the experiment.

The measure for subjective threat rating was initially adapted from a study by Bublatzky et al. (2019), where a visual analogue scale with the values 0 (*not at all threatening*) to 10 (*highly threatening*) was used. To increase sensitivity, this study used a rating scale of 0 to 100. The rating scale also included two other dimensions: “exciting” and “relaxing.” These were used to hide the aims of the study and were chosen based on the other emotional faces, which were happy and neutral, respectively. These two ratings were not analysed for this study as they are not related to the study’s aim to investigate threat perception.

#### Music Stimuli

Two types of auditory stimuli were used for this study: (a) “Insidious,” composed by Joseph Bishara for the 2010 horror film *Insidious*, served as the scary music condition. This soundtrack was chosen as horror film soundtracks are specifically designed to evoke feelings of fear in audiences (Gong & Zhang, 2021); and (b) white noise, which served as the control condition by providing an emotionally neutral sound for comparison (Niedenthal & Halberstadt, 2003). Following Logeswaran and Bhattacharya (2009), all auditory stimuli were edited into 15-s clips.

#### Face Stimuli

90 colour photographs (15 female and 15 male young adult models displaying happy, neutral, or angry expressions) were taken from the Tsinghua Facial Expression Database (Yang et al., 2020). Permission to use the database in this study was obtained. Each image was edited onto a grey background and presented in the centre of the laptop screen (1,920 × 1,080 resolution) with participants positioned 60 cm from the screen. At this distance, the total stimulus, which consisted of a grey background with a face stimulus in the center, subtended 18.46° in height and 32.08° in width. The face stimuli themselves subtended 12.08° in height and 9.05° in width. The order of presentation of faces was randomised.

### Design

A within-subjects experimental design was used for this study. Two factors were varied in the experimental design: music type (within subjects: scary music vs. white noise) by facial emotion (within subjects: angry vs. happy vs. neutral). The dependent variables were perceived threat ratings (0–100 scale) and pupil size (millimetres during the 1-s face presentation).

### Procedure

Participants attended the study at the Brain and Behaviour Research Unit lab at the authors’ institution. Participants first completed a basic demographic survey. Participants sat 60 cm from the screen and underwent a 9-point calibration for the eye tracker. The experiment was presented and run entirely in Tobii Studio 3.4.5, which handled both stimulus presentation and pupil data recording.

Each trial began with a 15-sd auditory stimulus (either horror music or white noise), during which participants fixated on a central point. This music presentation served as an effective prime, where the emotional context is established before the target stimulus evaluation. Immediately after the prime, a face (angry, happy, or neutral expression) appeared centrally for 1 s. Participants then rated the face on three dimensions (“threatening,” “exciting,” and “relaxing”) using a continuous scale from 0 (*not at all*) to 100 (*extremely*; see Figure 1). Importantly, the face stimuli were not displayed during the rating phase. This procedure aligns with the theoretical framework of affective priming, in which emotional context influences subsequent stimulus evaluation (Klauer & Musch, 2003).

**Figure 1.**
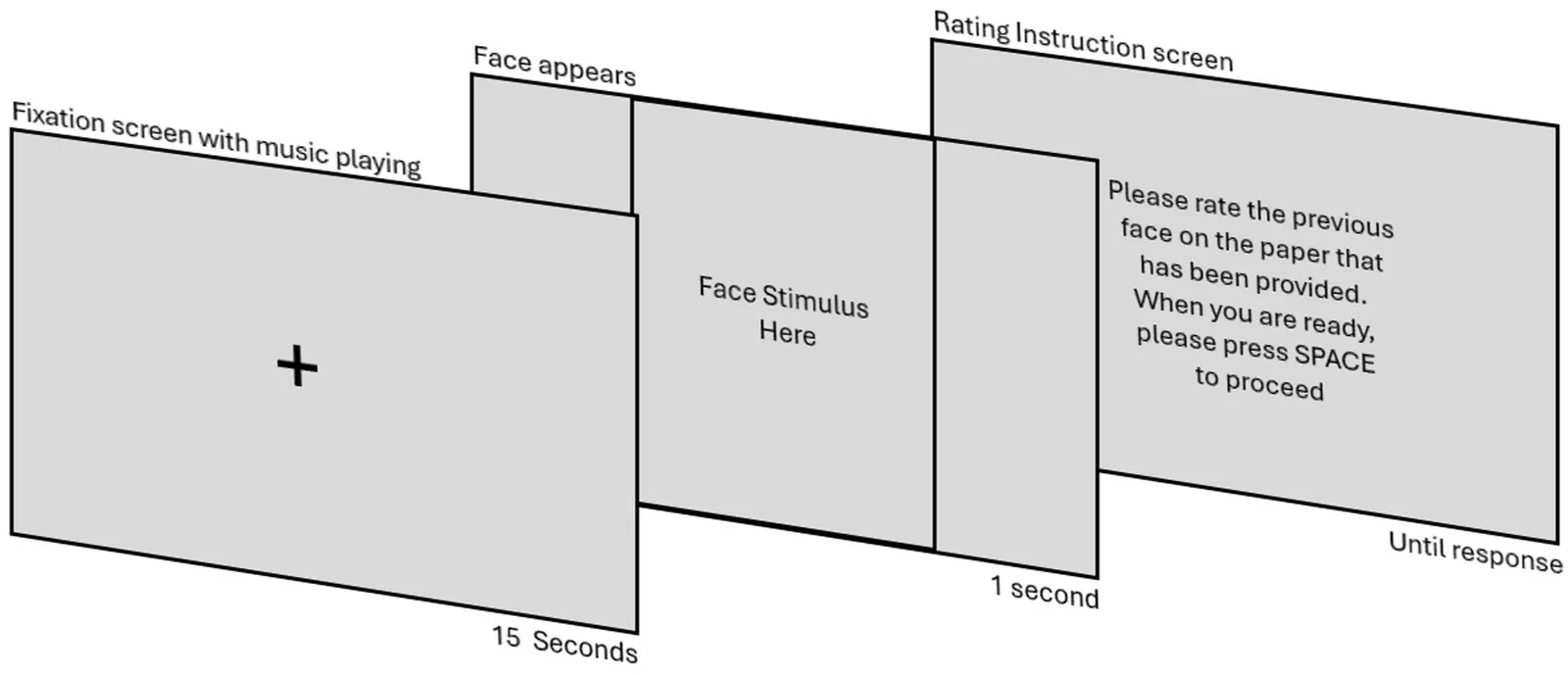
Trial structure.

Before the main experiment, participants completed two practice trials to ensure task comprehension. There were 90 experimental trials in total, with 15 trials per condition. The trials were randomised to minimise potential order effects and were presented in 3 blocks of 30 trials to reduce fatigue.

## Results

Descriptive statistics for threat ratings and pupil size across all conditions are shown in Table 1. For each condition (2 [music type] × 3 [facial type]), the threat rating across 15 trials for each participant was averaged, generating six averaged threat rating scores per participant.

**Table 1.** Descriptive Statistics for Threat Rating (*N* = 42) and Pupil Size (*N* = 45).

Music	Faces	Threat rating		Pupil size (mm)	
		*M*	*SD*	*M*	*SD*
Scary music	Angry	52.93	26.25	2.95	0.38
	Neutral	39.62	23.89	2.94	0.37
	Happy	32.40	27.56	2.92	0.39
White noise	Angry	34.92	21.71	2.95	0.38
	Neutral	14.18	10.07	2.91	0.39
	Happy	7.58	8.89	2.93	0.39

*Note. SD* = standard deviation.

Pupil size data were sampled continuously from both eyes at 60 Hz during the 1-s presentation of each facial stimulus, resulting in multiple data points per trial. These pupil size values were averaged across the 1-s exposure period in every trial, producing an average pupil size per trial. The 15 trial averages for each of the 6 conditions were then averaged again, producing 6 mean pupil size values per participant.

Data analysis was conducted in IBM SPSS Statistics (Version 28; International Business Machines Corporation). Prior to analysis, data were screened for outliers, defined as values more than ±3 standard deviations from the mean. For threat ratings, 3 outliers were identified and removed, resulting in a final sample of 42 participants for analyses. No outliers were detected for pupil size, and data from all 45 participants were retained for the analyses.

As shown in Table 1, participants’ threat ratings varied across the stimuli, ranging from 7 to 52 on a 0 to 100 scale. Unsurprisingly, angry faces were rated as the most threatening across both music conditions, but worth noting that threat ratings were modest, with scores mostly in the bottom two quartiles. To address the hypotheses, two separate 2 × 3 repeated measures ANOVAs were conducted to investigate the effect of music and emotional face type on participants’ subjective threat ratings and pupil size.

### Threat Ratings

The ANOVA on threat ratings revealed a significant main effect of music type, *F*(1, 41) = 44.80, *p* < .001, η_*p*_^2^ = .52. Threat ratings were higher in the scary music condition (*M* = 41.65, SE = 3.51) than in the white noise condition (*M* = 18.90, SE = 1.63). Thus, showing that exposure to scary music increases perceived threat for faces regardless of emotion type.

There was also a significant main effect of face type, *F*(1.42, 58.24) = 37.65, *p* < .001, η_*p*_² = .48 Bonferroni-corrected post hoc tests indicated that angry faces (*M* = 43.93, SE = 3.28) were rated as significantly more threatening than both neutral (*M* = 26.90, SE = 2.25, *p* < .001) and happy faces (*M* = 19.99, SE = 2.45, *p* < .001). Neutral faces were also rated as more threatening than happy faces (*p* = .003).

Most importantly, a significant interaction was found between music type and emotional face type, *F*(1.65, 67.59) = 6.40, *p* = .005, η_*p*_^2^ = .13, indicating a moderate effect, such that the effect of scary music on participants’ threat ratings was influenced by the emotional face type. Bonferroni-corrected pairwise comparisons (see Figure 2) showed that, in line with our first hypothesis, angry and neutral faces were rated as more threatening in the scary music condition (angry: *M* = 52.93, SE = 4.05; neutral: *M* = 39.62, SE = 3.69) compared to white noise (angry: *M* = 34.92, SE = 3.35; neutral: *M* = 14.18, SE = 1.554). However, contrary to our predictions that scary music would have little to no effect on happy faces, we found that happy faces were also rated as more threatening following scary music (*M* = 32.41, SE = 4.25) compared to white noise (*M* = 7.58, SE = 1.37).

**Figure 2.**
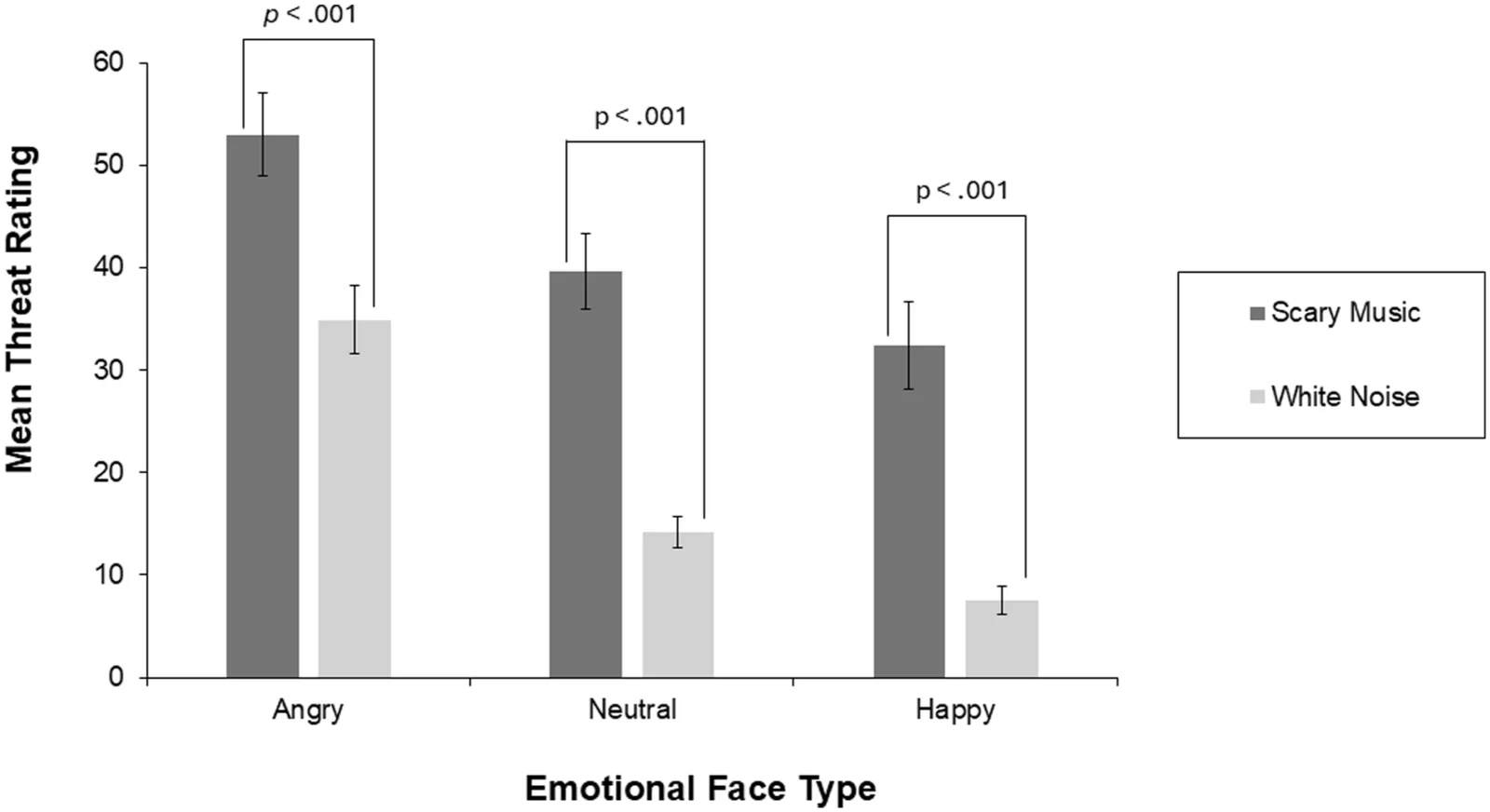
Effect of music type on threat ratings across emotional face types. *Note.* Error bars represent the standard error of the mean (SEM). SEM = standard error of the mean.

Further inspection of the interaction (see Figure 3) shows that in the white noise condition, the threat hierarchy was clear (angry > neutral > happy). In the scary music condition, this differentiation was reduced: angry faces remained most threatening, but neutral and happy faces no longer differed significantly. This suggests that scary music might amplify overall threat perception while blurring the distinction between neutral and happy expressions.

**Figure 3.**
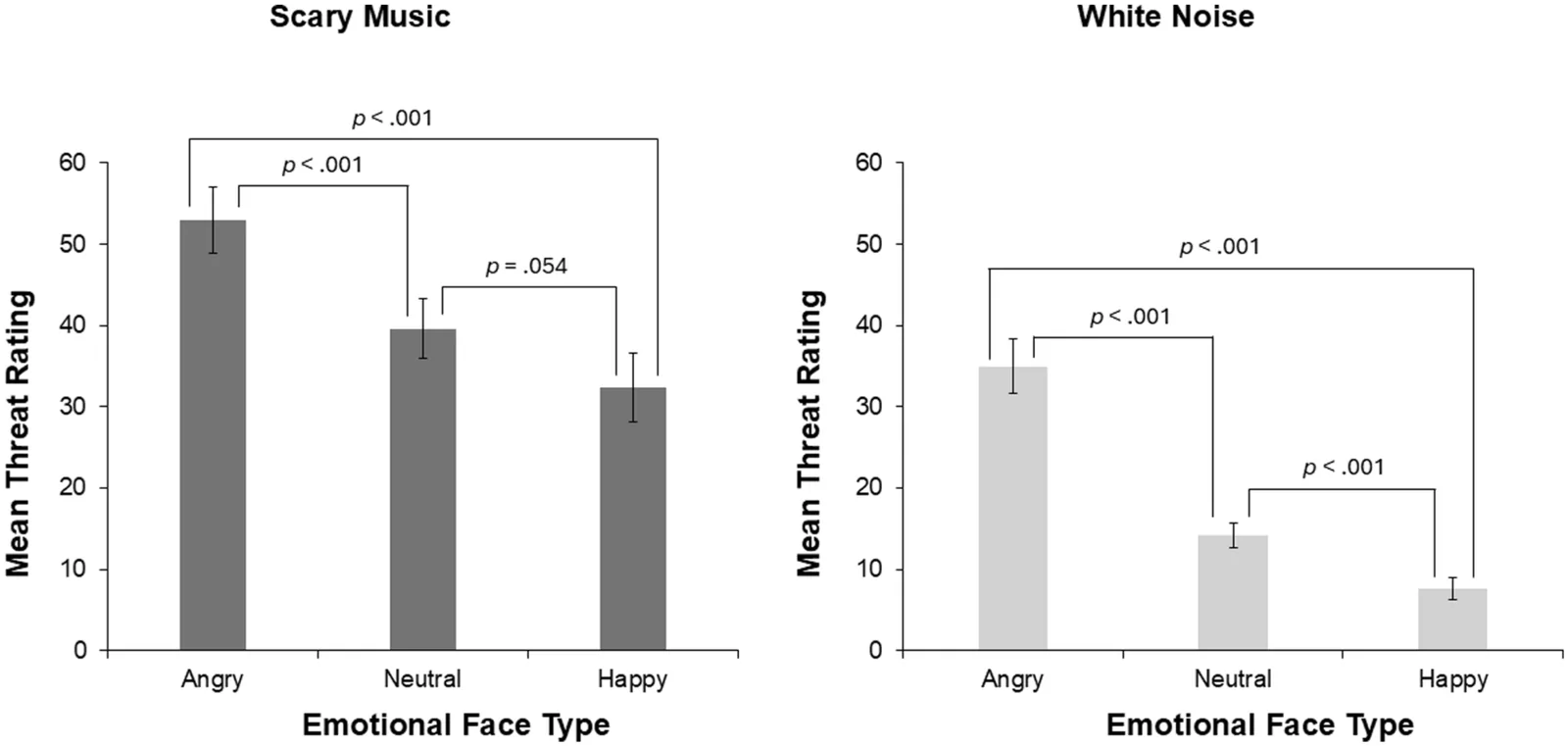
Interaction between music type and emotional face type on threat ratings. *Note.* Error bars represent the standard error of the mean (SEM). SEM = standard error of the mean.

### Pupil Size

The ANOVA on pupil size revealed no significant main effect of music type, *F*(1, 44) = 0.76, *p* = .387, η_*p*_^2^ *=* .017. Thus, contrary to our earlier prediction that scary music would generally increase pupil size, we found that pupil size in the scary music condition (*M* = 2.94, *SD* = 0.04) was not significantly larger than in the white noise condition (*M* = 2.93, *SD* = 0.04).

There was a significant main effect of face type, *F*(1.37,60.04) = 4.28, *p* = .031, η_*p*_² = .09. However, once Bonferroni corrections were applied to the post hoc tests, none of the pupil size differences between emotional face types were statistically significant (*p* > .05).

Interestingly, there was a significant interaction between music type and face type on pupil size, *F*(2, 88) = 3.17, *p* *=* .047, η_*p*_^2^ = .07, indicating that the effect of music type on pupil size was influenced by the emotional face type (see Figure 4). Specifically, Bonferroni-corrected pairwise comparisons show that pupil size was significantly larger for neutral faces following scary music (*M* = 2.94, SE = 0.05) compared to white noise (*M* = 2.91, SE = 0.06), whereas no significant music-related differences were observed for angry (scary music: *M* = 2.95, SE = 0.06; white noise: *M* = 2.95, SE = 0.06) or happy faces (scary music: *M* = 2.92, SE = 0.06; white noise: *M* = 2.93, SE = 0.06). We discuss this further below.

**Figure 4.**
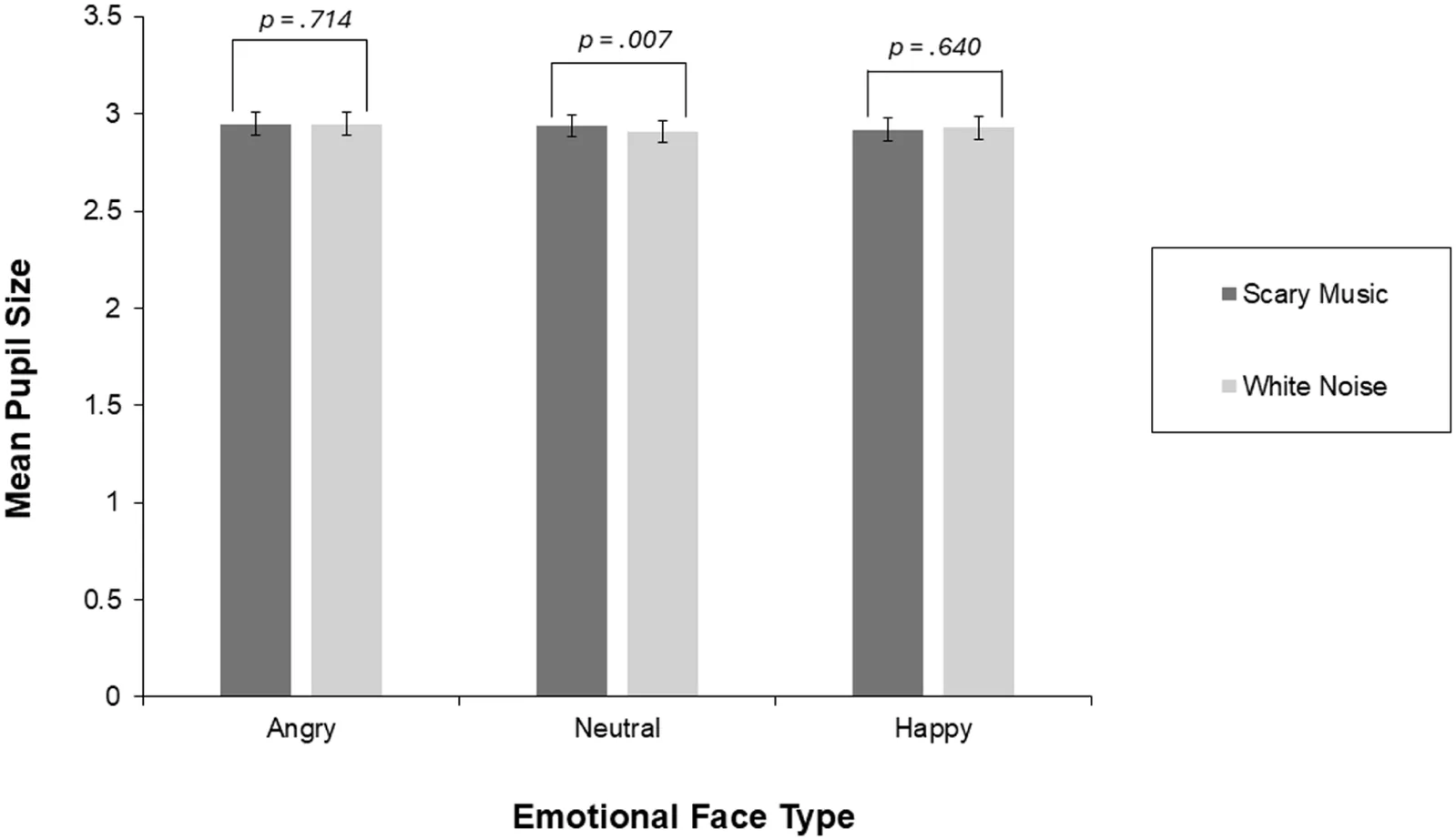
Effect of music type on pupil size across emotional face types. *Note.* Error bars represent the standard error of the mean (SEM). SEM = standard error of the mean.

## Discussion

The present study investigated the effect of scary music on individuals’ threat perception in various emotional faces (i.e., happy, neutral, and angry), using both subjective threat ratings and pupillometry. Overall, the results showed that scary music increased perceived threat across all facial expressions, with angry faces consistently rated as the most threatening. However, pupil responses did not show a robust global increase across conditions.

Participants’ threat ratings across all faces (regardless of emotion) were higher after listening to scary music than after listening to white noise. This finding aligns with Prinz and Seidel’s (2012) study, which similarly found that participants were more likely to report threatening interpretations of visual stimuli after listening to fearful-sounding music. In both the present study and that of Prinz and Seidel (2012), exposure to scary music was associated with an increase in participants’ threat perception, consistent with affective priming accounts (Klauer & Musch, 2003). The scary music may have acted as a negative affective prime, biasing participants’ subsequent perception of the faces in a similarly negative way. This suggests that the emotional, fear-inducing tone of the scary music is sufficient to act as an affective prime, shifting participants’ perception of all faces towards threat, even when the stimuli were not inherently threatening (as in the case of neutral and happy faces).

H1 predicted that scary music would elicit higher threat ratings specifically for angry and neutral faces, with little or no change for happy faces. Our results showed that while angry and neutral faces were rated as more threatening in the scary music condition, happy faces also received significantly higher threat ratings, to the point that happy and neutral faces were no longer clearly distinguishable. This contrasts with expectations that only angry and neutral faces would be affected, as neutral stimuli are often most susceptible to affective priming (Logeswaran & Bhattacharya, 2009). One possible explanation for this is emotional incongruence. The positive valence of happy faces may be undermined by their pairing with incongruent, fear-inducing music, creating an uncanny or “creepy” impression (Kjeldgaard-Christiansen & Clasen, 2023). This emotional incongruence from the auditory and visual stimuli may then distort the social meaning of happy expressions, making them less safe (i.e., more threatening). Moreover, smiles are not always reliable indicators of benevolence, as they can mask hostile or deceptive intentions (e.g., a duplicitous smile or sinister smile), which could further explain why happy faces appeared threatening in the scary music condition. Our findings offer a preliminary look at this effect, but establishing the exact nature of this shift remains for future work to address – specifically by investigating whether fear-inducing music leads listeners to attribute deceptive or sinister motives to happy facial expressions. Overall, these findings suggest that affective priming from negative auditory cues can extend their influence beyond threat-relevant or ambiguous stimuli (e.g., angry or neutral faces) to positive social signals.

Beyond subjective ratings, the present study examined pupil responses as a physiological index of autonomic arousal or salience to measure whether the effects of scary music were accompanied by corresponding physiological changes. Salient cues can engage subcortical salience-processing systems and trigger autonomic responses, leading to physiological changes such as pupil dilation, which is sensitive to emotionally arousing and salient stimuli (Einhäuser, 2017; Steinhauer et al., 2004). In this study, all visual stimuli were faces, which are inherently salient and emotionally arousing and may therefore have produced a relatively similar baseline level of arousal across trials. Within this context, variation in pupil responses is more likely to reflect differences in emotional content conveyed by facial expressions and background music, rather than general responses to faces per se. Accordingly, pupil dilation in the present study is interpreted as indexing heightened autonomic arousal associated with emotionally and motivationally salient facial and auditory cues.

Contrary to our prediction for H2, scary music did not significantly increase pupil size to emotional faces relative to white noise. This contrasts with prior work showing that negatively valenced stimuli can induce pupil dilation (Ansani et al., 2020; Partala & Surakka, 2003). One possibility is that although the scary music used in this study increased subjective threat perception, it may not have been sufficiently intense, or the task context may not have been conducive, to elicit a reliable change in pupil-linked autonomic arousal. This suggests that the bias in perception induced by scary music may operate primarily at a cognitive-perceptual level, without necessarily being accompanied by robust changes in pupil-based physiological arousal.

However, while scary music did not produce a global increase in pupil size, it selectively modulated responses depending on facial emotion, particularly increasing pupil size for neutral faces. This aligns with evidence that neutral stimuli are more susceptible to affective priming (Logeswaran & Bhattacharya, 2009). In the context of the current study, a plausible explanation is that both happy and angry faces are sufficiently arousing on their own, such that pupil dilation occurs in response to the emotional valence of the facial stimuli alone (Bradley et al., 2008). As a result, music-related effects may not have emerged because the pupillary response is already driven by the inherent emotional salience of these expressions. In contrast, neutral faces do not typically elicit such arousal, thereby providing room for the effects of music to be observed – specifically, greater pupil dilation with scary music due to its fear-inducing qualities. However, to confirm this explanation, we would need to directly test this by either systematically varying the arousal intensity of face stimuli or by including auditory-only and visual-only control blocks to disentangle whether music effects are overshadowed when paired with inherently arousing visual stimuli.

While the findings of this study offer useful insights into how music can influence the perception of threat, the outcome should be interpreted with caution because of ethnic considerations. In the absence of a suitable Malaysian face database at the time of data collection, a validated Chinese face database was used despite the participant sample being multiethnic. This may have introduced cross-ethnic biases in threat perception, as outgroup faces are sometimes judged more negatively (Ramiah et al., 2014). In addition, pupillometry studies have shown larger pupil responses to other-race faces, possibly reflecting greater cognitive effort required during processing and encoding (Goldinger et al., 2009; Wu et al., 2012). To control for these, future research should use ethically matched face stimuli that correspond to the participants’ backgrounds, or alternatively, include a balanced set of stimuli representing multiple ethnic groups.

Another limitation of the present study is that the sample was predominantly female, with only three male participants. Consequently, the observed effects of scary music on the threat perception of emotional faces may primarily reflect the characteristics of female participants. Prior research suggests that gender differences exist in emotional processing and threat sensitivity (Lambrecht et al., 2014; McClure et al., 2004), with anxiety-inducing music shown to elicit stronger physiological arousal in females than in males (Nater et al., 2006). This raises the possibility that males may respond differently to similar emotional priming effects. As a result, the generalizability of the present findings to male populations may be limited.

Building on the present findings, an important next step would be to examine whether music-induced changes in threat perception extend to social decision-making in more ecologically relevant contexts. Given that happy faces were perceived as more threatening when paired with scary music (potentially because of distorted interpretations of intent cues, such as a seemingly malicious smile), future work could employ a monetary trust game. In such a paradigm, participants could make financial decisions involving partners (confederates) whose faces (happy, neutral, and angry) are paired with scary versus neutral music. This approach would extend the current work beyond subjective ratings to behavioural economics, providing insight into how music influences real-world decision-making and social cooperation.

In conclusion, this study contributes to the literature on music and perception by providing novel insights demonstrating that scary music influences threat perception differently at both behavioural and physiological levels. Behavioural ratings showed a fairly robust affective priming effect of scary music across all emotional face types, while pupillometry revealed more selective changes to neutral faces in particular. These findings highlight how auditory context shapes social perception, and most importantly, underscore the value of combining subjective ratings and physiological measures to capture different facets of emotional processing.

Beyond the laboratory, the results provide implications for media contexts where music may bias perception of social information. For example, pairing scary music with images or footage of crime suspects in news reports, documentaries, or social media could lead viewers to perceive individuals as more threatening, regardless of the evidence presented. This raises important ethical questions about fairness and objectivity in media presentation to minimise bias.

## Data Availability

Data will be made available on request.
